# Neurogenetic interactions and aberrant behavioral co-morbidity of attention deficit hyperactivity disorder (ADHD): dispelling myths

**DOI:** 10.1186/1742-4682-2-50

**Published:** 2005-12-23

**Authors:** David E Comings, Thomas JH Chen, Kenneth Blum, Julie F Mengucci, Seth H Blum, Brian Meshkin

**Affiliations:** 1 Director, Carlsbad Science Foundation, Emeritus Professor City of Hope Medical Center, Duarte, California, USA; 2 Changhua Christian Hospital, Taiwan, Republic Of China; 3 Wake Forest University School Of Medicine, Department Physiology & Pharmacology, Medical Center Boulevard, Winston -Salem, North Carolina, Salugen, Inc. San Diego, California, USA; 4 Synapatmine, Inc., San Antonio, Texas, USA; 5 Salugen, Inc., San Diego, California, USA

**Keywords:** ADHD, attention, hyperactivity, inattention, genetics, aberrant behavioral co-morbidity, treatment, genomics

## Abstract

**Background:**

Attention Deficit Hyperactivity Disorder, commonly referred to as ADHD, is a common, complex, predominately genetic but highly treatable disorder, which in its more severe form has such a profound effect on brain function that every aspect of the life of an affected individual may be permanently compromised. Despite the broad base of scientific investigation over the past 50 years supporting this statement, there are still many misconceptions about ADHD. These include believing the disorder does not exist, that all children have symptoms of ADHD, that if it does exist it is grossly over-diagnosed and over-treated, and that the treatment is dangerous and leads to a propensity to drug addiction. Since most misconceptions contain elements of truth, where does the reality lie?

**Results:**

We have reviewed the literature to evaluate some of the claims and counter-claims. The evidence suggests that ADHD is primarily a polygenic disorder involving at least 50 genes, including those encoding enzymes of neurotransmitter metabolism, neurotransmitter transporters and receptors. Because of its polygenic nature, ADHD is often accompanied by other behavioral abnormalities. It is present in adults as well as children, but in itself it does not necessarily impair function in adult life; associated disorders, however, may do so. A range of treatment options is reviewed and the mechanisms responsible for the efficacy of standard drug treatments are considered.

**Conclusion:**

The genes so far implicated in ADHD account for only part of the total picture. Identification of the remaining genes and characterization of their interactions is likely to establish ADHD firmly as a biological disorder and to lead to better methods of diagnosis and treatment.

## Prevalence

ADHD is one of the most well-recognized childhood developmental problems. This condition is characterized by inattention, hyperactivity and impulsiveness. It is now known that these symptoms continue as problems into adulthood for 60% of children with ADHD. That translates into 4% of the US adult population, or 8 million adults. However, few ADHD adults are identified or treated. Adults with ADHD may have difficulty following directions, remembering information, concentrating, organizing tasks or completing work within time limits. If these difficulties are not managed appropriately, they can cause associated behavioral, emotional, social, vocational and academic problems. ADHD afflicts 3% to 7.5% of school-age children [1-4]. An estimated 30% to 70% of those will maintain the disorder into adulthood. Prevalence rates for ADHD in adults are not as well determined as rates for children, but fall in the 1% to 5% range. ADHD affects males at higher rate than females in childhood, but this ratio seems to even out by adulthood.

## Dispelling the myths

### How is ADHD diagnosed?

The diagnosis of ADHD is based on criteria outlined by the Diagnostic and Statistical Manual of the American Psychiatric Association Version 4-TR [1]. This is referred to as the DSM-IV-TR™. Table 1 illustrates these criteria. Several similar criteria were set out in earlier versions of the DSM. While the names have changed somewhat, all have included the letters ADD in one form or another, representing the core of the disorder – Attention Deficit Disorder. The subtypes in the DMS-IV are ADHD-I, representing predominately the inattentive type, ADHD-H, representing predominately the hyperactive-impulsive type, and ADHD-C, representing the combined type.

**Table 1:** DSM-IV Diagnostic Criteria for Attention-Deficit/Hyperactivity Disorder

A. Either (1) or (2)
(1) six (or more) of the following symptoms of **inattention **have persisted for at least 6 months to a degree that is maladaptive and inconsistent with developmental level:
*Inattention*
(a) often fails to give close attention to details or makes careless mistakes in schoolwork, work, or other activities (b) often has difficulty sustaining attention in tasks or play activities (c) often does not seem to listen when spoken to directly (d) often does not follow through on instructions and fails to finish schoolwork, chores, or duties in the workplace (not due to oppositional behavior or failure to understand instructions) (e) often has difficulty organizing tasks and activities (f) often avoids, dislikes, or is reluctant to engage in tasks that require sustained mental effort (such as schoolwork or homework) (g) often loses things necessary for tasks or activities (e.g., toys, school assignments, pencils, books, or tools) (h) is often easily distracted by extraneous stimuli (i) is often forgetful in daily activities
(2) six (or more) of the following symptoms of **hyperactivity-impulsivity **have persisted for at least 6 months to a degree that is maladaptive and inconsistent with developmental level:
*Hyperactivity*
(a) often fidgets with hands or feet or squirms in seat (b) often leaves seat in classroom or in other situations in which remaining seated is expected (c) often runs about or climbs excessively in situations in which it is inappropriate (in adolescents or adults, may be limited to subjective feelings of restlessness) (d) often has difficulty playing or engaging in leisure activities quietly (e) is often "on the go" or often acts as if "driven by a motor" (f) often talks excessively
*Impulsivity*
(g) often blurts out answers before questions have been completed (h) often has difficulty awaiting turn (i) often interrupts or intrudes on others (e.g., butts into conversations or games)
B. Some hyperactivity-impulsive or inattentive symptoms that caused impairment were present before age 7 years
C. Some impairment from the symptoms is present in two or more settings (e.g., at school [or work] and at home)
D. There must be clear evidence of clinically significant impairment in social, academic, or occupational functioning
E. The symptoms do not occur exclusively during the course of a Pervasive Developmental Disorder, Schizophrenia, or other Psychotic Disorder and are not better accounted for by other mental disorder (e.g., Mood Disorder, Anxiety Disorder, Dissociative Disorder, or a Personality Disorder).
*Code *based on type:
**314.01 Attention-Deficit/Hyperactivity Disorder, Combined Type: **if both Criteria A1 and A2 are met for the past 6 months
**314.00 Attention-Deficit/Hyperactivity Disorder, Predominately Inattentive Type: **if Criterion A1 is met but Criterion A2 is not met for the past 6 months
**314.01 Attention-Deficit/Hyperactivity Disorder, Predominately Hyperactive-Impulsive Type: **if Criterion A2 is met but Criterion A1 is not met for the past 6 months

### ADHD is a common disorder

Estimates of the frequency of the various types of ADHD, based on population surveys, have shown variable results. The advantage of population based samples, in contrast to clinic based samples, is that individuals in the community who have not sought medical attention are included. Table 2 shows the results of Wolraich et al. [3] for all three subtypes of ADHD based on teacher reports for grades K through 5 in a countywide sample of 4,323 children in Tennessee. An epidemiological study of children and adolescent twins in Missouri showed a frequency of all types of ADHD of 3.5% in girls and 7.5% in boys [2]. In the Wolraich et al study [3], only 11 to 33% of the cases had received a diagnosis of ADHD and only 8 to 26% were being treated with stimulant medication. A Centers for Disease Control survey showed that in the year 2003 4.4 million children 4 to 17 years of age were reported to have a diagnosis of ADHD. Of these, 56% were receiving medications for the disorder [5]. These figures are contrary to the notion that ADHD is over-diagnosed and over-treated. While many of these children can be handled by appropriate teaching methods and do not require treatment, the figures suggest that ADHD-I, at least, is probably under-diagnosed and under-treated.

**Table 2:** Prevalence of various types of ADHD in the general population

From Wolraich et al. (1998)	
Hyperactive/Impulsive	2.6
Inattentive	8.8
Combined	4.7
Total	16.1
	M/F ratio 4:1

### Clinical aspects of ADHD

It is one thing to read a list of the symptoms in Table 1 and quite another to experience the ADHD child at first hand, as teachers and parents of affected children do. Individuals with ADHD tend to be disorganized. Children have messy lockers and rooms and both children and adults have cluttered desks. Their daily activities tend to be chaotic. They have trouble making plans and even more trouble in carrying out plans in an orderly fashion. Because of problems with attention and focus, they have trouble completing what they start and leave tasks unfinished, plans unrealized. Attics and basements are likely to be filled with partly completed projects, repairs, and notebooks; desk drawers are likely to be cluttered with unfinished letters, outlines and project plans. Although many individuals with ADHD are highly intelligent, they tend to be underachievers, a result of their poor concentration and inability to sustain interest. They become bored easily and have trouble entertaining themselves. Reading books is very difficult. Family, friends, teachers and coworkers often become impatient with them and expect them to fail. Their life is so full of tumult that even a minor additional change in their routine can be upsetting.

Individuals with ADHD have a very low level of tolerance to frustration and stress. This results in irritability and poor anger control. The anger tends to come on suddenly and explosively with slamming doors, punching holes in walls, verbal abuse of those around them, tantrums, and leaving important meetings in a frenzy. Children get into fights, adults blow up and lose jobs and alienate friends. Afterwards they are sorry, but the damage is done. Because of their low tolerance for frustration they are very impatient. They hate to wait in line, and delays of any kind make them frantic. Whatever is going on – a trip, a movie, a class, a discussion – they want it to go quickly and be finished. Because of their impulsivity both children and adults may leap into action without thinking of consequences. As adults, they drive too fast, use power tools carelessly, and plunge into activities without thinking of the danger. As children they often appear fearless, do dangerous things, climb too high in trees, and may dart into traffic without looking. The result is they often hurt themselves or others. People with ADHD have trouble with their orientation to time and space. They may have to stop and think which is their right hand and which is their left; may have difficulty following a set of instructions, reading a map or telling time. People often complain they can never get to places on time. Because of their difficulty in planning ahead, they leave too little time to get places. If they live 30 minutes from the place of an appointment, they often leave home at the time of the appointment, making themselves 30 minutes late. It takes little imagination to realize that may of these traits make for difficult interpersonal relationships and problems in school and on the job. With adults a history of many failed marriages and many job changes is common. This is a flavor of just some of the issues that ADHD children and adults face.

### ADHD is a genetic disorder

For many years, clinicians caring for children with ADHD have noted that the condition is common in one or both of the parents. While this suggests that ADHD may have a strong genetic basis, environmental factors could cause the same familiar pattern. Twin studies provide much stronger evidence for the role of genetic factors. Several large twin studies of ADHD have been completed in the last 15 years. They show that the concordance rate in identical twins is usually greater than 65% while that in fraternal twins is usually less than 40%. This is consistent with 75 to 95% of ADHD being genetically caused, the remainder being environmental [6-8]. One reason why twin studies are valuable is that if a behavior or disorder was primarily environmental, the effect should be comparable whether the twins were identical for fraternal, since both identical and fraternal twins usually live together for at least the early part of their life. A significant drop in concordance rate from identical to fraternal twins suggests genetic but not environmental factors.

The environmental portion can be divided into a shared and unshared component. The shared component refers to exposure to the same environment while the unshared portion refers to exposure to different environments. The former is more likely to occur early in childhood, while the latter is more likely to occur later in childhood. The early shared environment is the part of life that Freud and many other psychiatrists and psychologists assumed was the most formative part of a child's life in terms of their adult behavior. An additional part of the twin studies of great interest was that the shared environment was usually found to contribute to essentially 0% of the environmental component [6]. Most of the environmental component was due to unshared experiences later in life.

A number of adoption studies of ADHD have also been reported [9-11]. These are particularly valuable in separating genetic from environmental factors. If a child is adopted at birth, there is no opportunity for the biological parent to influence the behavior. If a child develops a disorder such as ADHD, and studies show that the biological father but not the adopting father had ADHD, this is especially strong evidence of the role of genetic factors. This is the conclusion reached from the ADHD adoption studies.

### ADHD is a polygenic disorder

When diseases or disorders or traits are due to genetic factors, there are several mechanisms by which they can inherited. Such conditions can essentially be divided into single gene disorders and polygenic disorders. Single gene disorders include hemophilia, cystic fibrosis, neurofibromatosis and Huntington disease. In single gene disorders a rare mutation results in the complete disruption of the function of a gene. Some of the greatest advances in genetics during the past 100 years have come from the elucidation of the genes for virtually every single gene disorder. Their DNA has been cloned, sequenced and the gene localized to a specific chromosomal region.

Polygenic disorders, by contrast, are due to the interactive or epistatic effects of many different genes on different chromosomes, each gene contributing to only a small part of the picture (variance). These genes interact with environmental factors. Except for a few rare families [12], all behavioral disorders such as manic-depressive disorder, schizophrenia, major depression, panic disorder, autism and ADHD [3] are likely to be polygenic. While we do not yet know the total number of genes involved, it is likely to range from 50 to several hundred. In contrast to the gene defects for single gene disorders (mutations), the defects for polygenic disorders are much less severe, otherwise they would be single gene disorders. Thus, we call them gene variants instead of gene mutations, and individuals have to inherit a number of them if they are to cause a clinical effect [13]. A second distinction is that mutations that severely affect gene function are very rare. Since they are often present in less than 1 in 100,000 individuals the diseases they cause are also very rare. In fact, all single gene disorders combined affect less than 1.5% of the population. By contrast, the gene variants involved in polygenic disorders are common and polygenic disorders themselves are common. This "common gene, common disorder" theory of polygenic disorders has gained wide acceptance. An alternative theory, of "rare gene, common disorder," postulates a large number of rare mutations of different genes [14].

In association studies of a wide range of behavioral disorders, even when the association is significant the percent of the variance attributable to that gene is usually in the 0.5 to 3% range and averages less than 1.5%. This suggests that even if genes only account for 72 to 95% of the total variance, 50 or more different genes would be involved [15-17]. This does not mean that every affected individual has inherited 50 or more of these variants. It is likely that only a subset of the total potential set of gene variants is required in a given individual. Because of this, polygenic disorders show a great deal of genetic heterogeneity [17]. That is, different individuals with ADHD are likely to have inherited somewhat different sets of genes. However, each affected ADHD individual must have inherited enough gene variants to pass a liability threshold, allowing them to develop ADHD.

### ADHD is a spectrum disorder

**Figure 1:**
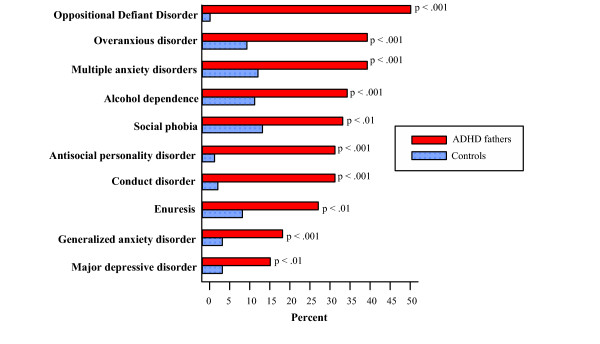
Comorbid disorders in ADHD from Biederman et al, 1993 [82].

It has been known for many years that if an individual inherits enough genes to develop any given behavioral disorder, their risk of developing a second behavioral disorder is 2 to 4 times greater than for the general population. This is probably because different behavioral disorders share some gene variants. Thus, the more a person exceeds the required threshold number of gene variants, the greater the likelihood they will develop more than one behavioral problem, thus the term spectrum disorders. Some of the most common coexisting or comorbid spectrum disorders seen in individuals with ADHD are oppositional defiant disorder, conduct disorder, major depressive disorder, anxiety disorders, obsessive compulsive disorder, bipolar disorder, learning disorders, and substance abuse disorder including alcoholism and drug addiction. The frequency of some of these disorders is illustrated in Figure 1[4]. This shows the spectrum disorders seen in the fathers of children with ADHD. Since these fathers had not sought medical care, this type of study avoids the biases inherent in a study of a clinic sample. The most likely explanation for the presence of spectrum disorders is that they share some genes in common, as well as some genes unique to each disorder [15,16].

### ADHD and many other complex disorders represent the upper end of a continuum of severity

After viewing the DSM-IV criteria for the diagnosis of ADHD, one of the most commonly voiced objections is, "every child has some of those symptoms." As with every other polygenic trait, ADHD symptoms lie on a continuum of severity. This is true of height, weight, IQ, blood pressure, cholesterol level, depression, dyslexia, anxiety and may other characteristics. These traits follow a bell-shaped curve of magnitude or severity. Many children have too few symptoms to meet the criteria. They may be somewhat inattentive or hyperactive at times but they do not meet all the criteria of ADHD. Note that 6 or more symptoms must be present, must meet the qualifiers of severity in the diagnostic criteria (almost everything is "often" not "occasionally") they must be present for 6 months or more and must be maladaptive and inconsistent with the normal developmental level. Because of these qualifiers some individuals may barely meet the criteria and are sufficiently mild not to require treatment. Others, however, are at the extreme end of the bell-shaped curve and are so symptomatic that everyone coming into even brief contact with them can suspect the diagnosis. Physicians arbitrarily pick a cut-off point for many diseases or disorders. Those on the extreme end of the curve have the disorder, those with less extreme symptoms do not. This may give a false illusion of a dichotomous trait. For example, the diagnosis of hypertension is usually based on a consistent diastolic blood pressure of 90 mmHg or more. Some individuals have severe life threatening hypertension with a diastolic blood pressure consistently above 120 mmHg while others have mild hypertension where the diastolic blood pressure is sometimes normal and sometimes too high. Because it is a continuum does not mean hypertension does not exist.

An even better example is depression. Everyone is occasionally depressed. This does not mean the diagnosis of major depression is invalid or worthless because everyone can relate to it. Some are so depressed they sleep all the time, can't get out of bed, eat poorly, lose or gain weight, have zero libido, are suicidal and desperately need treatment. Ironically, even some professionals who clearly understand that major depression is a real entity but lies on a continuum of severity may have trouble understanding that the same is true of ADHD. The cut-off point is set to secure help for those with symptoms severe enough to interfere with their lives, and leave those with minimal symptoms with no diagnosis.

### ADHD has lifelong effects

One of the most common misconceptions about ADHD is that it goes away by the time an individual is a young adult. In California, this is written into the MediCal law. Stimulants are no longer covered for the treatment of ADHD in adults because it is assumed the disorder is gone by that time. One feature leading to this misconception is that motor hyperactivity often does decrease with age. However, there is much less decrease in inattention, and if individuals are rated on a global assessment of functioning, there is little improvement with age [4].

### Stimulus hypersensitivity and "overload."

While stimulus overload is especially characteristic of children with autism, many children with ADHD are also very sensitive to sound, sight, smell or other sensory inputs [18]. Awareness of this helps parents and teachers to understand poor attention in large, noisy school classrooms. Reports suggest that some ADHD children are responding so intensely to environmental stimuli ignored by other children that their experience is comparable to trying to talk on a cell phone in a crowded, noisy barroom. As one teacher reported, for example, a boy diagnosed with ADHD told her, at the end of class on his first day of Ritalin treatment: "It's wonderful: now I can hear you."

### Learning primarily with visual images

Two pathways relevant to learning are that linking the linguistic cortex to the hippocampus and that for remembering visual images, which links the visual cortex to the hippocampus via the dorsolateral medial entorhinal cortex [19]. The latter pathway is essential for spatial orientation and tracking, and spatial memory – as in how we remember where our car is parked. Some ADHD children have difficulty with this skill. Others have difficulties with the linguistic memory pathway. This is typified by ADHD children with comorbid dyslexia who are unable to make images of written words that link the auditory linguistic cortex to memory. They might, for example, have difficulty distinguishing between the spelling "otehr" and "other."

### ADHD versus ADHD + conduct disorder

There have been a number of longitudinal studies of ADHD in which a group of individuals diagnosed in childhood were followed for a number of years to assess how they performed as adults [20-23]. In many of these studies the outcome was poor, with significant increases in substance abuse, trouble with the law, difficult interpersonal relationships and problems with employment. This has given the impression that all ADHD children have a bad outcome. However, when some of these studies were carefully analyzed, or when the study itself was appropriately designed, it became apparent that if the cases are divided into those with ADHD only and those with ADHD + conduct disorder (CD), it was the ADHD + CD cases that had poor outcomes while the ADHD only individuals often had outcomes that were not markedly different from those of normal children. This is consistent with the many studies in the past 50 years that have shown that one of the most stable of all diagnoses in psychiatry is CD [24-27]. On average, 50% of children diagnosed as conduct disorder still had symptoms of CD, or its adult equivalent, antisocial personality disorder (ASPD), 5 to 25 years later [24-28]. The DMS-IV-TR criteria for conduct disorder are given in Table 3. CD is present in 25 to 40% of ADHD children. Up to 25 percent of male prison inmates have ADHD + ASPD [29]. Treatment of ADHD in a prison population results in improved behavior and lower recidivism rates if the treatment is continued after release [30].

**Table 3:** DSM-IV Criteria of Conduct Disorder

A. A repetitive and persistent pattern of behavior in which the basic rights of other or major age-appropriate societal norms or rule are violated, as manifested by the presence of three (or more) of the following criteria in the past 12 months, with at least one criterion present in the past 6 months.
**Aggression to people and animals**
(1) often bullies, threatens, or intimidates others (2) often initiates physical fights (3) has used a weapon that can cause serious physical harm to others (e.g. a bat, brick, broken bottle, knife, gun) (4) has been physically cruel to people (5) has been physically cruel to animals (6) has stolen while confronting a victim (e.g., mugging, purse snatching, extortion, armed robbery) (7) has forced someone into sexual activity
**Destruction of property**
(8) has deliberately engaged in fire setting with the intention of causing serious damage (9) has deliberately destroyed others' property (other than by fire setting)
**Deceitfulness or theft**
(10) has broken into someone else's house, building, or car (11) often lies to obtain goods or favors or to avoid obligations (i.e., "cons" others) (12) has stolen items of nontrivial value without confronting a victim (e.g., shoplifting, but without breaking and entering; forgery)
**Serious violations of rules**
(13) often stays out at night despite parental prohibitions, beginning before age 13 years (14) has run away from home overnight at least once while living in parental or parental surrogate home (or once without returning for a lengthy period) (15) is often truant from school, beginning before age 13 years
B. The disturbance in behavior causes clinically significant impairment in social, academic, or occupational functioning
C. If the individual is age 18 years or older, criteria are not met for Antisocial Personality Disorder
Specify type based on age at onset:
**Childhood-Onset Type: **onset of at least one criteria characteristic of Conduct Disorder prior to age 10 years
**Adolescent-Onset Type: **absence of any criteria characteristic of Conduct Disorder prior to age 10 years
**Specify severity: **mild (few criteria met), moderate and severe (many criteria met)

### ADHD + ODD

A second disorder commonly comorbid with ADHD is oppositional defiant disorder (ODD). The DMS-IV criteria for ODD are given in Table 4. It is the ODD rather than the ADHD symptoms that most often drive parents to distraction. An interesting aspect of ODD is that it is often site specific. Thus, many children only present with ODD symptoms in the home, and often direct the behavior at their mother. Some children with severe ODD at home can be angels at school. It is likely that they control their tantrums and talking back at school because peer pressure prevents them from making fools of themselves in front of others. They have no such restraint at home. ODD is present in 40 to 60% of children with ADHD [31,32]. The most credible explanation for why these two disorders, and other comorbid disorders, are so common in ADHD is that they share many genes in common [16].

**Table 4:** DSM-IV Criteria of Oppositional Defiant Disorder

A. A pattern of negativistic, hostile, and defiant behavior lasting at least 6 months, during which four (or more) of the following are present:
(1) often loses temper (2) often argues with adults (3) often actively defies or refuses to comply with adult's requests or rules (4) often deliberately annoys people (5) often blames others for his or her mistakes or misbehavior (6) is often touchy or easily annoyed by others (7) is often angry and resentful (8) is often spiteful or vindictive
Note: Consider a criterion met only if the behavior occurs more frequently than is typically observed in individuals of comparable age and developmental level.
B. The disturbance in behavior causes clinically significant impairment in social, academic, or occupational functioning.
C. The behaviors do not occur exclusively during the course of a Psychotic or Mood Disorder.
D. Criteria are not met for Conduct Disorder, and if the individual is aged18 years or older, criteria are not met for Antisocial Personality Disorder.

### ADHD has a lifelong effect on function

**Figure 2:**
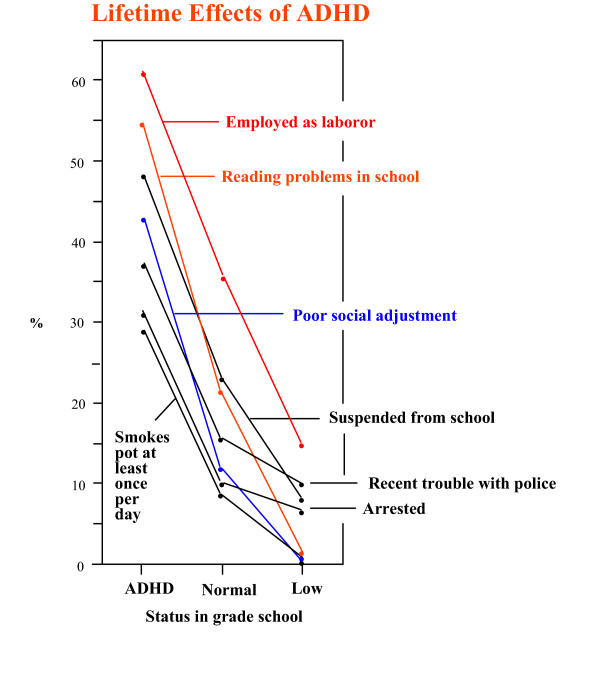
Longitudinal studies of children with low, intermediate and high ADHD scores in early grade school, from Howell et al: Pediatrics 76:185–190, 1985 [22].

Having pointed out that much of the poor outcome in ADHD children is due to the comorbid presence of CD, we would like to present the 1985 study by Howell and coworkers [22]. While this longitudinal study did not distinguish between ADHD and ADHD + CD, it did something no other study has done: it compared the outcomes of three groups of children instead of just ADHD children and controls. Children in the early grade school years were evaluated on a continuum of ADHD symptoms and divided into three groups, those scoring in the highest 10% (ADHD group), those in the lowest 10% (low ADHD group), and the rest ("normal") group. They were then re-evaluated after they graduated from high school. The remarkable finding was that in virtually every aspect of life the low ADHD group performed best, the normals were intermediate and the ADHD group performed worst (Figure 2). This should not be taken to suggest that children with ADHD always underachieve. Again, we wish to emphasize there are many examples in which the restless, workaholic, always-have-to-be-doing-something, I-need-to-be-my-own-boss, characteristics of ADHD subjects result in very successful lives. Thus, in the right combination, some of the symptoms we have been discussing in a negative light can be used to great advantage.

### ADHD is a disorder of prefrontal lobe function

Many of the symptoms of ADHD parallel the symptoms of individuals with destructive lesions of the prefrontal lobes [33-35]. When the dorsolateral portions of the prefrontal lobes are affected by traumatic injury there is impaired attention, distractibility, disinhibition of behavior, poor long term planning, impulsivity, lack of motivation, poor abstract reasoning, poor executive functioning and poor organizational skills, all of which are present in ADHD individuals. By contrast, lesions of the orbitomedial portion of the frontal lobe are associated with aggression, emotional outbursts, poor self-control, lack of guilt, empathy or remorse, and anti-social and psychopathic behavior [36]. These are the symptoms typical of CD and antisocial personality disorder. Thus, the finding that ADHD is a genetic disorder suggests the defective genes involved cause a dysfunction of the prefrontal lobes. As discussed below, one of the brain neurotransmitters likely to be involved in causing this dysfunction is dopamine.

### Some ADHD is a disorder of parietal lobe function

In some children with ADHD, especially those with learning disorders, the parietal lobes are also likely to be involved [37]. Studies of children with both ADHD and reading or other learning disabilities indicated they had abnormally high levels of norepinephrine breakdown products [38]. Norepinephrine is the arousal neurotransmitter of the brain, associated with waking the brain up in the morning and setting it to an optimal level of arousal. Studies in animals suggest that both too much and too little norepinephrine can be associated with hyperactivity [39]. The parietal lobes also carry some of the centers for speech and language as well as an area for attention. Thus, it is not surprising that defects in the parietal lobes can be associated with ADHD combined with learning disorders.

### ADHD and substance abuse

As mentioned above, two of the common comorbid spectrum disorders in ADHD are alcoholism and drug abuse [4044]. The reward pathways of the brain are also located in the frontal lobes and limbic system. They provide pleasure for a number of behaviors that are critical to the continued existence of the individual and the species, such as eating and having sex. These are termed natural rewards. The reward pathways are rich in dopamine carrying neurons and it is the release of dopamine that produces the feelings of pleasure. In addition to food and sex, all drugs of abuse result in the release of dopamine in the reward pathways. This is responsible for the feelings of euphoria or the high that these drugs produce. These are termed unnatural rewards. We have previously proposed a Reward Deficiency Syndrome (RDS) [45] suggesting that genetic variants in dopamine genes result in defective functioning of the reward system such that individuals with these defects are much more likely to seek out additional stimulation of their reward pathways by turning to drugs, alcohol, excessive sexual activity and risk-taking activities such as hang gliding and bungie jumping. In this regard, Lee *et al*. [46] found an association between novelty seeking (NS) and both the dopamine D4 receptor gene (DRD4) long alleles and the *Taq1 A1 *and *Taq1 B1 *sites of the dopamine D2 receptor gene. The term *Taq*1 refers to the type of restriction endonuclease that cuts a DNA sequence at a specific site. These results therefore confirmed previous findings in which the long repeats of the DRD4 polymorphism were related to NS personality trait, and suggested that the less frequent DRD2 alleles were also associated with the reward -dependent trait [47].

### ADHD and pathological gambling

Different neurological studies have found pathological gamblers to have high impulsivity and poor performance in tasks involving frontal/executive functions. These symptoms are similarly observed with individuals diagnosed with ADHD. It is noteworthy that pathological gambling is an addiction that is not confounded by the problems of ingesting a drug. PET studies of individuals engaged in video poker have documented a release of dopamine in the striatum [48]. We found that 50.9 %of 171 pathological gamblers carried the D_2_A1 allele compared to 25.9% of the 714 known non-Hispanic Caucasian controls screened to exclude drug and alcohol abuse; p <0.000001, odds ratio = 2.96 [49]. The *DRD2 *gene was associated with severity in that the D_2_A1 allele was present in 63.8% of those in the upper half of severity (odds ratio versus controls = 5.03) compared to 40.9% in the lower half of severity. Of those who had no comorbid substance abuse, 44.1% carried the D_2_A1 allele, compared to 60.5% of those who had comorbid substance abuse. These results suggest that genetic variants at the *DRD2 *gene play a role in pathological gambling and support the concept that variants of this gene are a risk factor for impulsive and addictive behaviors 49

In this regard, others have utilized questionnaires, structured interviews and behavioral tasks to evaluate the history of ADHD in pathological gamblers. It was found that pathological gamblers scored higher on the Barratt Impulsivity Scale (BIS) than controls, but only those with a history of childhood ADHD showed a greater impulsivity in behavioral tasks [50].

As discussed below, it has often been claimed that the treatment of ADHD with stimulants will encourage children to abuse drugs when they are older. It is much more likely that ADHD and substance abuse share many gene variants in common, and share a deficiency of dopamine in the frontal lobe and limbic system, and this is the reason ADHD and especially ADHD + CD children are also at risk for substance abuse. The best way to prevent substance abuse is to ensure that the ADHD and the resulting frontal and parietal lobe dysfunctions are adequately treated so these individuals do not turn to street drugs to self-medicate. Studies suggest that the prevalence of substance abuse disorder is lower in ADHD children who are adequately treated with stimulants than in those who are untreated [51].

### What genes are involved?

It is one thing to know that a disorder is largely genetic and another to know which genes are involved, especially if the disorder is polygenically inherited. Because each gene contributes to only a small part of the total picture, the gene variants involved can be difficult to identify. The most powerful method is the use of association studies. These studies compare the frequency of gene variants (polymorphisms) in individuals with and without ADHD, or examine whether certain gene variants are preferentially transmitted to a child with ADHD. The genes that have been examined in ADHD and related behavioral disorders tend to be in three classes: enzymes, receptors and transporters, especially those that involve neurotransmitters. Binding to the post-synaptic cell occurs at receptors that are specific for a given transmitter. In addition to being broken down by enzymes, the neurotransmitters can be cleared from the synapse by transporters that return the neurotransmitters to the pre-synaptic cell. As with the receptors, the transporters are neurotransmitter-specific. Perhaps the best known example is the serotonin transporter. Drugs that inhibit the action of the serotonin transporter are called selective serotonin re-uptake inhibitors or SSRIs.

There are an estimated 25,000 human genes. More efficient methods for testing large numbers of genes in association studies are rapidly being developed. Until these techniques are fully developed and relatively inexpensive, we perforce examine the genes (candidate genes) that we feel are most likely to be involved. To date, variations at a number of candidate genes have shown a significant association with ADHD. These include genes for three dopamine receptors *(DRD2, DRD4, DRD5)*, the dopamine transporter *(DAT1 or SLC6A3)*, the serotonin transporter *(HTT or SLC6A4)*, norepinephrine receptors *(ADRA2A, ADRA2C)*, the norepinephrine transporter *(NET or SLC6A2)*, and others [15,16,52]. Of these, we will only discuss the dopamine transporter in more detail because it has relevance to ADHD in both humans and animals, the association has been replicated in a number of studies, and it has important implications for understanding the mechanism of action of the stimulants and possibly for identifying which individuals are most likely to respond to treatment.

The most frequently examined variant in the dopamine transporter is a 40 base pair repeat polymorphism. The most common variants or alleles are called 9 and 10. A number of studies have shown that the 10 allele is more common in Caucasians with ADHD. Brain imaging studies show an increased availability of the dopamine transporter in ADHD subjects compared to controls across all age groups [53]. It is especially interesting that these studies have also shown that the treatment of ADHD subjects with methyphenidate (Ritalin) decreases the level of the dopamine transporter availability back to normal or below normal levels [54]. In contrast to the association of ADHD in humans with elevated DAT1 levels, knockout mice that have no *DAT1 *gene are also very hyperactive [55]. While this may seem to contradict the human studies, a number of compensatory effects can occur when a gene is totally eliminated at conception. In addition, both too much and too little of a neurotransmitter may have similar effects in many systems. Some preliminary studies have suggested a differential response of methylphenidate in those who carry different alleles of the *DAT1 *gene [56]. Furthermore, it has been suggested [56] that children possessing the 10 repeat VNTR allele of the DAT1 gene might be particularly responsive to methylphenidate because of its DAT-blocking action. Others have further examined this hypothesis. In a sample of 119 Irish children, Killey et al. [57] also reported that the 10 repeat allele predicted a positive clinical outcome. Not all studies have found this association, but it begins to open the way to predicting drug response by genetic testing before the drugs are given.

A number of genome scans, using sibling-pair linkage techniques for identifying relevant genes, have been performed [58,59]. A number of chromosomal sites showing significant but modest linkage have been identified. There is agreement between studies about some areas and disagreement about others. Association studies will have to be performed to identify the specific genes in the areas responsible for the positive signals.

Because the *DAT1 *and other genes contribute to only a modest part of the total picture, a more powerful method of examining the genetics of ADHD may be to test the interactive (epistatic) effects of multiple genes. Preliminary studies simultaneously examining variants at 42 different genes indicate the importance of norepinephrine genes in the etiology of ADHD [16]. These studies, still in their infancy, may point the way toward understanding the complex genetics of ADHD and its comorbid conditions, identifying genetic subtypes of ADHD, and identifying before any drugs are given who will respond to which drugs and who may have undesirable side effects. Since there is some overlap with SUD in many ADHD adults, the possible role of other dopaminergic genes and regulators should not be overlooked [60-65].

### Treatment of ADHD: Why use stimulants? How do they work?

Many double blind studies over the past 40 years have agreed that stimulants such as methylphenidate, dextro-amphetamine, and others are very effective in the treatment of 70–80 % of children and adults with ADHD. One of the myths of ADHD is that ADHD children show a paradoxical effect of being calmed by stimulants while "normal" individuals are stimulated by them. However, studies have shown that activity levels are decreased and attention levels are increased by stimulants in individuals with and without ADHD. The difference is that since the levels of hyperactivity and inattention are much higher in ADHD subjects, the improvement is relatively much greater, giving the impression that they respond while non-ADHD subjects do not.

**Figure 3:**
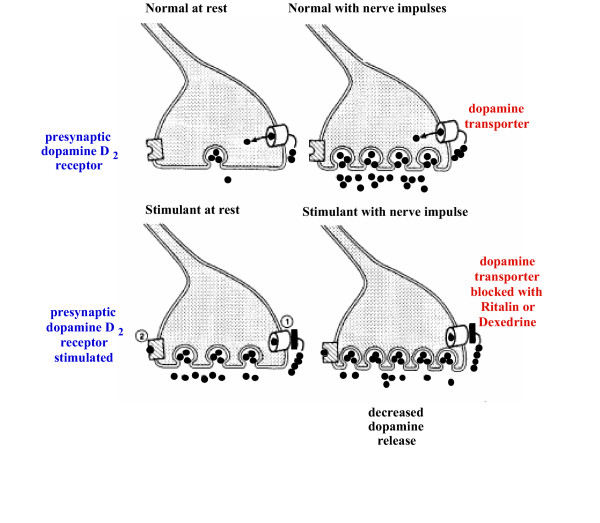
Dexedrine mode of action. From Seeman and Madras: Mol. Psychiatry 3:386–396,1998 [83].

How do stimulants work? It is known that, as in the effect of SSRIs on the serotonin transporters, the stimulants inhibit dopamine transporters (and norepinephrine transporters). Since hyperactivity is related to excessive dopamine activity in the basal ganglia, this would seem on the face of it to make things worse instead of better. However, Figure 3 illustrates the stimulants work in ADHD. Figure 3a shows the basal, unstimulated state with dopamine stored in the vesicles and low levels of dopamine in the synapse. Figure 3b shows the result of stimulation of the dopamine neuron, with the vesicles releasing dopamine into the synapse and re-uptake of the dopamine into the presynaptic neuron by the dopamine transporters. Figure 3c shows that in the presence of stimulants, the function of the dopamine transporters is partially blocked and the basal level of dopamine increases in the synapse. This results in occupation of the presynaptic dopamine D_2 _receptors. When the nerve is stimulated (Figure 3d), the amount of dopamine released from the vesicles is deceased because of the occupation of the presynaptic D_2 _receptors. This results in a decrease in dopaminergic stimulation in the basal ganglia, where the density of the D_2 _receptors is the highest. Of particular interest, there are few D_2 _receptors in the prefrontal lobe. Thus, dopamine activity in the prefrontal lobes is increased instead of decreased. This is consistent with a model of ADHD in which there is too little dopamine in the frontal lobes, hence the symptoms of prefrontal lobe deficits, and too much dopamine in the basal ganglia, hence motor hyperactivity and, not infrequently, motor tics [66]. The stimulants correct both the prefrontal lobe deficiency and the basal ganglion excess of dopamine.

Despite this indication of how well-suited stimulant medications are to the treatment of ADHD, many still worry that our children are receiving a form of "speed." Studies have shown that in order to obtain a "high", stimulants need to reach the brain very quickly. This requires intravenous or nasal administration, or the use of doses that exceed therapeutic recommendations. At therapeutic oral doses the stimulants used for treatment of ADHD do not cause a euphoric high. Perhaps the best indicator of this is that one of the hardest parts of the treatment of ADHD children is to get them to take their medication; they do not come begging for more. This, however, is no guarantee that these drugs are never abused. It is important that children and adolescents with ADHD should not have free access to their medications, since it is clear that these drugs can be abused when given nasally, or intravenously or in high doses. Keeping track of the medications helps to ensure that they are not sold for illicit use. There is also the potential problem of inducing aggressive behavior in those individuals possessing the DRD2 *Taq A1 *allele [67].

A second class of medications that work primarily on norepinephrine pathways, such as clonidine, guanifacine, and atomoxetine, can also be quite effective. Clonidine and guanifacine are especially useful in treating individuals with both ADHD and chronic tics (Tourette syndrome) since clonidine and guanifacine uniquely treat both these conditions [68]. Physicians are often reluctant to treat individuals with ADHD and Tourette syndrome with stimulants for fear of exacerbating the tics. However, consistent with the above mechanism of action of stimulants, significant exacerbation is unusual and often the tics are unchanged or improved following stimulant treatment [69].

### Treatment of ADHD comorbid disorders

As indicated above, it is often the comorbid disorders such as ODD and CD that cause the greatest distress to parents of children with ADHD. In our experience, the atypical neuroleptics such as risperidone, olanzipine and molindone can be very effective in the treatment of these comorbid conditions.

### Alternative treatments

Because of their concern about the use of medications, many parents seek alternative methods of treatment of ADHD. Most clinicians agree that a combination of medication and behavioral modification is the most effective approach to the treatment of ADHD, even though the medications contribute the most [70]. Children with ADHD may also respond well to adjustments in education such as those provided by an Individual Education Plan (IEP). The following additional alternatives are among the most often used.

### Family therapy and education

Family therapy relates to therapy that involves every member of the family. This can be especially effective since there are often many issues that affect the entire family. These involve educating the parents, siblings and affected child that ADHD is a genetic-biochemical disorder and is not the child's fault. This does not mean that ADHD children should not be held responsible for their behavior. It does mean that the level of discipline needs to be modulated by an understanding of the biological basis of the disorder. Family therapy can also address the frequent concern of siblings that the misbehaving child often gets a disproportionate degree of attention. Many other issues relating to having a child with ADHD in the family benefit from family therapy and parent education.

#### EEG biofeedback

EEG biofeedback usually utilizes feedback from a game played on a TV screen to attempt by training to alter the levels of alpha, beta and delta waves in the brain. It has the advantage that no drugs are used and it appears to be effective in some cases [71]. The disadvantages are that it can be expensive; satisfactory double-blind testing of its effectiveness has been very difficult; and the effects may not be long-lasting. However, others have found success with EEG neurofeedback training for ADHD in a clinical setting [71].

#### Herbal remedies

A large number of herbal remedies have been used by ADHD patients. Sometimes they seem to be effective, sometimes not, or their effectiveness may be short-lived. Many parents turn to them because they are perceived as "natural." However, to be effective they must contain an active ingredient and the identity of this ingredient is usually not known. In addition, a wide range of other ingredients may be present that are not necessary or may cause undesirable side effects [72].

#### Neutriceuticals

In contrast to herbal remedies, the compositions of neutriceuticals are precisely known. They usually consist of amino acids, vitamins, minerals and other known compounds. Because they are closer to food substances than drugs, they do not have such rigorous FDA restrictions as drugs and can be purchased over the counter. Because a number of amino acids have direct or indirect effects on the levels of specific neurotransmitters, they have the potential for helping to control some of the symptoms of ADHD. Neutriceuticals have the advantage that double-blind studies can be easily carried out. It is not unlikely that some combinations of the above compounds, carefully tested in double-blind studies, may play a supporting role in controlling some of the symptoms of ADHD [73-75].

### Heavy metal toxicity: Role in autism and ADHD

The frequencies of both autism and ADHD have appeared to increase in the last decade or more. There has been particular emphasis on the possibility that the presence of mercury in vaccines might play a role in this increase. These issues have been reviewed in detail by Kirby [76], including the potential interaction between heavy metals and specific polymorphisms that may increase an individual's susceptibility to toxins. Because of this concern, mercury has now been removed from all vaccines in the United States; it was removed from vaccines in Sweden and Denmark after 1993. Despite this, the frequency of autism has either remained high or continued to increase [77]. This lack of association with mercury in vaccines is consistent with twin studies that show a very high heritability for autism, thus implying minimal influence from environmental factors. Mercury in vaccines has also been implicated in the etiology of ADHD. While data on the effect of removal of mercury from vaccines on the incidence of ADHD in Sweden and Denmark are not available, the results for autism, and the high heritability of ADHD, suggest that mercury poisoning is probably not a major factor. A role of other heavy metals such as lead and manganese has also been suggested. It is difficult entirely to rule out a role of heavy metal poisoning in ADHD, and further studies are indicated. One of the appeals of the theory that exposure to toxic substances that are new to the environment may cause autism and ADHD is that it may explain the increased frequencies of these disorders. This is based in part on the assumption that increases in frequency could not be due to increases in the frequency of ADHD genes because gene variants do not increase in frequency rapidly. One of us has suggested a mechanism by which these genes could have rapidly increased in frequency. This idea proposes that two factors unique to the latter part of the 20^th ^century could have played such a role. These are the availability of effective birth control in the 1960s, and the dramatic increase in the percentage of individuals attending college since the Second World War. Individuals carrying genes for ADHD and learning disorders are less likely to go on to college and post graduate work and more likely to start a family in their early 20 s. Individuals who do not carry these genes are more likely to attend college and put off childbearing into their late 20 s or early 30 s. This would result in a more rapid increase in the first set of genes. Whether this would be sufficient to account for an increase in frequency of ADHD and autism must await studies of the frequency of a range of genes by birth cohort.

## Future perspective of genetic research

At the 4th International meeting of the Attention Deficit Hyperactivity Disorder Molecular Genetics Network, chaired by Stephen V Farone, investigators from around the world convened in May 2002 at Harvard Medical School and presented their findings about genes and ADHD. The putative ADHD genes with pooled odds ratios greater than 1.0 included the following: DAT1 = 1.3; DRD4 = 1.4; DRD5 = 1.5; and 5HT1B = 1.5. It was concluded that although technological and statistical advances in molecular genetics have allowed researchers to begin to identify the association of specific genes with ADHD, continued collaborative efforts are needed to elucidate the genetic underpinnings of this complex phenotype fully [76]. Since this time, numerous other loci have been shown to be associated with ADHD including calcyon (DRD11P) [78], SNAP25, serotonin transporter, dopamine β-hydroxylase, monamine oxidase A [79], tryptophan hydroxylase 2 [80], TPH2 [80] several norepinephrine genes, BDNF, and others. Because of the small percentage of the variance attributed to each gene, multiple additional studies will be required to determine which of these will be multiply replicated [81]. In the future, testing for a panel of genes may lead to improved diagnosis and treatment of ADHD.

## Summary

ADHD is a common, complex, polygenic genetic, but highly treatable disorder that can have a profound lifelong effect on brain function. The genes currently identified as playing a causative role in ADHD account for only a small percentage of the total picture. The identification of additional ADHD, CD and ODD genes, and their interactions, holds the promise of firmly establishing ADHD as a biological disorder and of identifying better methods of diagnosis and treatment, emphasizing the need for polypharmacy.

## List of abbreviations

**ADHD **Attention deficit hyperactivity disorder

ADHD-C ADHD combined type

ADHD-H ADHD hyperactive type

ADHD-I ADHD inattentive type

*ADRA2A *adrenergic A2a receptor gene

*ADRA2C *adrenergic 2C receptor gene

BDNF brain derived neurotrophic factor

CD Conduct disorder

DAT dopamine transporter

DAT1 Dopamine transporter Genetic Engineering News

DRD2 Dopamine D_2 _receptor gene

DRD4 Dopamine D_4 _receptor gene

DSM-IV-TR™ Diagnostic and Statistical Manual of the American Psychiatric Association -IV Text revision

FDA Federal Drug Administration

EEG electroencephalogram

HTT serotonin transporter gene

IQ Intelligence Quotient

NET norepinephrine transporter gene

ODD Oppositional defiant disorder

PET Positron emission tomography

RDS Reward Deficiency Syndrome

*SLC6A3 *Dopamine transporter gene

*SLC6A4 *serotonin transporter gene

*SLC6A2 *serotonin A2 receptor gene

SNAP25 synaptosomal-associated protein, 25kDa

SSRI Selective serotonin reuptake inhibitors

Taq I Taq I restriction endonuclease

TPH2 tryptophan hydroxylase 2

VNTR variable number tandem repeats

## Competing interests

The author(s) declare that they have no competing interests.

## Authors' contributions

**David Comings **major primary author

**Thomas JH Chen **– Co-Author

**Kenneth Blum- **Co-Author

**Julie Mengucci- **Prime editorial reviewer

**Seth H. Blum- **Stylistic References and review

**Brian Meshkin- **Co-editorial reviewer

## References

[B1] American Psychiatric Association (2000). Diagnostic and Statistical Manual of the American Psychiatric Assn IV-TR™.

[B2] Neuman RJ, Sitdhiraksa N, Reich W, Ji TH, Joyner CA, Sun LW, Todd RD (2005). Estimation of prevalence of DSM-IV and latent class-defined ADHD subtypes in a population-based sample of child and adolescent twins. Twin Res Hum Genet.

[B3] Wolraich ML, Hannah JN, Baumgaertel A, Feurer ID (1998). Examination of DSM-IV criteria for attention deficit/hyperactivity disorder in a county-wide sample. J Dev Behav Pediatr.

[B4] Biederman J, Mick E, Faraone SV (2000). Age-dependent decline of symptoms of attention deficit hyperactivity disorder: impact or remission definition and symptoms type. Am Psychiatry.

[B5] Centers for Disease Control and Prevention (CDC) (2005). Mental health in the United States. Prevalence of diagnosis and medication treatment for attention-deficit/hyperactivity disorder – United States, 2003. MMWR Morb Mortal Wkly Rep.

[B6] Sherman DK, McGue MK, Iacono WG (1997). Twin concordance for attention deficit hyperactivity disorder: a comparison of teachers' and mothers' reports. Am J Psychiatry.

[B7] Hudziak JJ, Derks EM, Althoff RR, Rettew DC, Boomsma DI (2005). The genetic and environmental contributions to attention deficit hyperactivity disorder as measured by the conners' rating scales – revised. Am J Psychiatry.

[B8] Kuntsi J, Rijsdijk F, Ronald A, Asherson P, Plomin R (2005). Genetic influences on the stability of attention-deficit/hyperactivity disorder symptoms from early to middle childhood. Biol Psychiatry.

[B9] Cadoret RJ, Stewart MA (1991). An adoption study of attention deficit/hyperactivity/aggression and their relationship to adult antisocial personality. Compr Psychiatry.

[B10] Cunningham L, Cadoret RJ, Loftus R, Edwards J (1995). Studies of adoptees from psychiatrically disturbed biological parents. I. Psychiatric conditions in childhood and adolescence. Br J Psychiatry.

[B11] Cantwell DP, Rosenthal D, Brill H (1975). Genetic studies of hyperactive children: Psychiatric illness in biologic and adopting parents. Genetic Research in Psychiatry.

[B12] Brunner HG, Nelen M, Breakefield XO, Ropers HH, van Oost BA (1993). Abnormal behavior associated with a point mutation in the structural gene for monoamine oxidase A. Science.

[B13] Comings DE, Blum K (1996). Polygenic inheritance of psychiatric disorders. Handbook of Psychiatric Genetics.

[B14] Pritchard JK (2001). Are rare variants responsible for susceptibility to complex diseases?. Am J Hum Genet.

[B15] Comings DE, Gade-Andavolu R, Gonzalez N, Wu S, Muhleman D, Blake H, Dietz G, Saucier G, MacMurray JP (2000). Comparison of the role of dopamine, serotonin, and noradrenaline genes in ADHD, ODD and conduct disorder: multivariate regression analysis of 20 genes. Clin Genet.

[B16] Comings DE, Gade-Andavolu R, Gonzalez N, Wu S, Muhleman D, Blake H, Chiu F, Wang E, Farwell K, Darakjy S, Baker R, Dietz G, Saucier G, MacMurray JP (2000). Multivariate analysis of associations of 42 genes in ADHD, ODD and conduct disorder. Clin Genet.

[B17] Comings DE (2003). The Real Problem with Association Studies. Am J Med Genet (Neuropsychiatric Genetics).

[B18] Miller D, Blum K (2000). Overload Attention Deficit Disorder and the Addictive Brain.

[B19] Hafting T, Fyhn M, Molden S, Moser M, Moser EI (2005). Microstructure of a spatial map in the entorhinal cortex. Nature.

[B20] Mannuzza S, Klein RG, Bessler A, Malloy P, LaPadula M (1998). Adult psychiatric status of hyperactive boys grown up. Am J Psychiatry.

[B21] Hechtman L, Weiss G (1986). Controlled prospective fifteen-year follow-up of hyperactives as adults. Non-medical drug and alcohol use and anti-social behavior. Can J Psychiatry.

[B22] Howell DC, Huessy HR (1981). Hyperkinetic behavior followed from 7 to 21 years of age. Strategic Interventions for Hyperactive Children.

[B23] Klein R, Mannuzza S (1991). Long-term outcome of hyperactive children: A review. J Am Acad Child Adolesc Psychiatry.

[B24] Olweus D (1979). Stability of aggressive reaction patterns in males: A review. Psychological Bull.

[B25] Robins LN (1966). Deviant Children Grown Up.

[B26] Farrington DP, Loeber R, Elliott DS, Hawkins JD, Kandel DB (1990). Advancing knowledge abut the onset of delinquency and crime. Adv Clin Child Psychology.

[B27] McCord J (1979). Some child-rearing antecedents of criminal behavior in adult men. J Pers Soc Psychol.

[B28] Graham P, Rutter M (1973). Psychiatric disorders in the young adolescent: A follow-up study. Proc Roy Soc Med.

[B29] Eyestone L, Howell RJ (1994). An epidemiological study of attention-deficit disorder and major depression in a male prison population. Bull Am Acad Psychiatry Law.

[B30] McCallon D Personal communication.

[B31] Biederman J (1987). High rate of affective disorders in probands with attention deficitdisorder and their relatives: A controlled family study. Am J Psychiatry.

[B32] Barkley RA, Fischer M, Edelbrock CS, Smallish L (1990). The adolescent outcome of hyperactive children diagnosed by research criteria: I. An 8-year prospective follow-up study. J Am Acad Child Adolesc Psychiatry.

[B33] Spencer TJ, Biederman J, Wilens TE, Faraone SV (2002). Overview and neurobiology of attention-deficit/hyperactivity disorder. J Clin Psychiatry.

[B34] Castellanos FX (1997). Toward a pathophysiology of attention-deficit/hyperactivity disorder. Clin Pediatr (Phila).

[B35] Barkley RA (1997). Attention-deficit/hyperactivity disorder, self-regulation, and time: toward a more comprehensive theory. J Dev Behav Pediatr.

[B36] Damasio AR (1994). Descartes' Error.

[B37] Posner MI, Peterson SE (1990). The attention system of the human brain. Annu Rev Neurosci.

[B38] Halperin JM, Newcorn JH, Koda VH, Pick L, McKay KE, Knott P (1997). Noradrenergic mechanisms in ADHD children with and without reading disabilities: A replication and extension. J Am Acad Child Adolesc Psychiatry.

[B39] Aston-Jones G, Cohen D (2005). An integrative theory of locus coeruleus-norepinephrine function: adaptive gain and optimal performance. Annu Rev Neurosci.

[B40] Goodwin DW, Schulsinger F, Hermansen L, Guze SB, Winokur G (1975). Alcoholism and the hyperactive child syndrome. J Nerv Ment Dis.

[B41] Loney J, Kramer J, Milich R, Gadow K, Loney J (1981). The hyperkinetic child grows up: Predictors of symptoms, delinquency, and achievement at follow-up. Psychosocial Aspects of Drug Treatment for Hyperactivity.

[B42] Wilens TE, Biederman J, Mick E, Faraone SV, Spencer T (1997). Attention deficit hyperactivity disorder (ADHD) is associated with early onset substance use disorder. J Nerv Ment Dis.

[B43] Wood DR, Wender PH, Reimherr FE (1983). The prevalence of attention deficit disorder, residual type, or minimal brain dysfunction, in a population of male alcoholic patients. Am J Psychiatry.

[B44] Horner RB, Scheibe KE (1997). Prevalence and implication of attention-deficit hyperactivity disorder among adolescents in treatment for substance abuse. J Amer Acad Child Adolesc Psychiat.

[B45] Blum K, Cull JG, Braverman ER, Comings DE (1996). Reward Deficiency Syndrome. American Scientist.

[B46] Lee H-J, Lee H-S, Kim Y-K, Kim L, Lee MS, Jung I-K, Suh K-Y, Kim S (2003). D2 and D4 dopamine Receptor Gene Polymorphisms and Personality Traits in a young Korean Population. American J Med Gen.

[B47] Noble EP, Ozkaragoz TZ, Ritchie T, Zhang X, Belin TR, Sparkes RS (1998). D2 and D4 dopamine receptor polymorphisms and personality. American J Med Gen.

[B48] Koepp MJ, Gunn RN, Lawrence AD, Cunningham VJ, Dagher A, Jones T, Brooks DJ, Bench CJ, Grasby PM (1998). Evidence for striatal dopamine release during a video game. Nature.

[B49] Comings DE, Rosenthal RJ, Lesieur HR, Rugle L, Muhleman D, Chiu C, Dietz G, Gade R (1996). A study of the dopamine D2 receptor genin pathological gambling. Pharmacogenetics.

[B50] Rodriguez-Jimenez R, Avila C, Ponce G, Ibanez MI, Jimenez M, Monasor R, Jurado R, Jimenez-Arriero MA, Rubio G, Palomo T (2005). Neuropsychology of pathological gamblers: influence of attention-deficit/ hyperactivity disorder in childhood. International meeting on implications of comorbidity for etiology and treatment of neuropsychiatric disorders.

[B51] Biederman J, Wilens T, Mick E, Spencer T, Faraone SV (1999). Pharmacology of attention-deficit/hyperactivity disorder reduces risk for substance use disorder. Pediatrics.

[B52] Blum K, Chen JTH, Blum S, Downs WB, Braverman Mengucci J, Meshkin B Nutrigenomics & pharmacogenomics: A scientific wonderland. Biology Of Social Life.

[B53] Dougherty DD, Bonab AA, Spencer TJ, Rauch SL, Madras BK, Fischman AJ (1999). Dopamine transporter density in patients with attention deficit hyperactivity disorder. Lancet.

[B54] Dresel S, Krause J, Krause KH, LaFougere C, Brinkbaumer K, Kung HF, Hahn K, Tatsch K (2000). Attention deficit hyperactivity disorder: binding of [99mTc]TRODAT-1 to the dopamine transporter before and after methylphenidate treatment. Eur J Nucl Med.

[B55] Giros BSR, Wightman RM, Caron MG (1996). Hyperlocomotion and indifference to cocaine and amphetamine in mice lacking dopamine transporter. Nature.

[B56] Winsberg B, Comings D (1999). Association of the dopamine transporter gene (DAT1) with poor methylphenidate response. J Am Acad Child Adolesc Psychiatry.

[B57] Kirley A, Lowe N, Hawi Z, Mullins C, Daly G, Waldman I, McCarron M, O'Donnell D, Fitzgerald M, Gill M (2003). Association of the 480 bp DAT1 Allele With Methyphenidate Response in a Sample of Irish Children With ADHD. Am J Med Gen.

[B58] Blum K, Noble EP, Sheridan PJ, Montgomery A, Ritchie T, Jagadeeswaran P, Nogami H, Briggs AH, Cohn JB (1990). Allelic association of human dopamine D_2 _receptor gene in alcoholism. JAMA.

[B59] Blum K, Noble EP, Sheridan PJ, Finley O, Montgomery A, Ritchie T, Ozkaragoz T, Fitch RJ, Sadlack F, Sheffield D (1991). Association of the A1 allele of the D2 dopamine receptor gene with severe alcoholism. Alcohol.

[B60] O'Hara BF, Smith SS, Bird G, Persico AM, Suarez BK, Cutting GR, Uhl GR (1993). Dopamine D2 receptor RFLPs, haplotypes and their association with substance use in black and Caucasian research volunteers. Hum Hered.

[B61] Goldman D, Brown GL, Albaugh B, Robin R, Goodson S, Trunzo M, Akhtar L, Lucas-Derse S, Long J, Linnoila M (1993). DRD2 dopamine receptor genotype, linkage diseqilibrium and alcoholism in American Indians and other populations. Alcohol Clin Exp Res.

[B62] George SR, Cheng R, Nguyen T, Israel Y, O'Dowd BF (1993). Polymorphisms of the D_4 _dopamine receptor alleles in chronic alcoholism. Biochem Biophys Res Commun.

[B63] Neville MJ, Johnstone EC, Walton RT (2004). Identification and Characterization of ANKK1: A novel kinase gene closely linked to DRD2 on chromosome band 11q23.1. Human Mutation.

[B64] Comings DE, Wasserstein J, Wolf L, LeFevre FF (2001). The clinical and molecular genetics of ADHD and Tourette syndrome: Two related polygenic disorders. Adult Attention Deficit Disorder: Brain Mechanisms and Life Outcomes.

[B65] Chen TJH, Blum K, Mathews D, Fisher L, Schnautz N, Braverman ER, Schoolfield J, Downs BW, Comings DE (2005). Are dopaminergic genes involved in a predisposition to pathological aggression? Hypothesizing the importance of "super controls" in psychiatricgenetic research of complex behavioral disorders. Medical Hypotheses.

[B66] Comings DE, Rakel RE, Bope ET (2003). Treatment of Tourette Syndrome,. Coon's Current Therapy 2003.

[B67] Gadow KD, Nolan EE, Sverd J (1992). Methylphenidate in hyperactive boys with comorbid tic disorder: II. Short-term behavioral effects in school setting. J Am Acad Child Adolesc Psychiatry.

[B68] Geyer MA (2005). The family of sensorimotor gating disorders: Comorbidities or diagnostic overlaps?. International meeting on implications of comorbidity for etiology and treatment of neuropsychiatric disorders.

[B69] The MTA Cooperative Group (1999). Multimodal Treatment Study of Children with ADHD. A 14-month randomized clinical trial of treatment strategies for attention-deficit/hyperactivity disorder. Arch Gen Psychiatry.

[B70] Lubar JF, Swartwood MO, Swartwood JN, O'Donnell PH (1995). Evaluation of the effectiveness of EEG neurofeedback training for ADGD in a clinical setting by changes in T.O.V.A. scores., behavioral ratings and WISC-R performance. Biofeedback and Self Regulation.

[B71] Brown R, Gerbarg Pl, Ramazanov Z (2002). Rhodiola rosea: A phytomedicinal overview. J Amer Botanical Council.

[B72] Defrance JF, Hymel C, Trachtenberg MC, Ginsberg LD, Schweitzer FC, Estes S, Chen TJH, Braverman ER, Cull JG, Blum K (1997). Enhancement of attention processing by Kantroll in healthy humans. A pilot study. Clin Electroenceph.

[B73] Wood DR, Reimer FW, Wender PH (1985). Treatment of Attention deficit Disorder with DL-phenylalanine. Psychiatry Research.

[B74] Chen TJH, Blum K, Payte JT, Schoolfield J, Hopper D, Stanford M, Braverman ER (2004). Narcotic antagonists in drug dependence: pilot study showing enhancement of compliance with SYN-10, amino-acid precursors and enkephalinase inhibition therapy. Medical Hypotheses.

[B75] Kirby D (2005). Evidence of Harm.

[B76] Stehr-Green T, Tull P, Stellfeld M, Mortenson PB, Simpson D (2003). Autism and Thimerosal-Containing Vaccines. Lack of Consistent Evidence for an Association. Amer J Preventive Med.

[B77] Faraone S (2003). Report from the 4th International Meeting of the Attention Deficit Hyperactivity Disorder Molecular Genetics Network. American J Med Gen.

[B78] Laurin N, Misener VL, Crosbie J, Ickowicz A, Pathare T, Roberts W, Malone M, Tannock R, Schachar R, Kennedy JL, Barr CL Association of the calcyon gene (DRD1IP) with attention deficit/hyperactivity disorder. Mol Psychiatry.

[B79] Paclt I, Koudelova J, Krepelova A, Uhlikova P, Gazdikova M, Bauer P Biochemical markers and genetic research of ADHD. Review. Neuro Endocrinol Lett.

[B80] Walitza S, Renner TJ, Dempfle A, Konrad K, Wewetzer C, Halbach A, Herpertz-Dahlmann B, Remschmidt H, Smidt J, Linder M, Flierl L, Knolker U, Friedel S, Schafer H, Gross C, Hebebrand J, Warnke A, Lesch KP Transmission disequilibrium of polymorphic variants in the tryptophan hydroxylase-2 gene in attention-deficit/hyperactivity disorder. Mol Psychiatry.

[B81] Comings DE, Blum K (2005). Reward Deficiency Syndrome: Genetic Aspects Of Behavioral Disorders and Validation. International meeting on implications of comorbidity for etiology and treatment of neuropsychiatric disorders.

[B82] Seeman P, Madras BK (1998). Anti-hyperactivity medication: methylphenidate and amphetamine. Molecular Psychiatry.

[B83] Biederman J, Faraone SV, Spencer T, Wilens T, Norman D, Lapey KA, Mick E, Lehman BK, Doyle A (1993). Patterns of psychiatric comorbidity, cognition, and psychosocial functioning in adults with attention deficit hyperactivity disorder. Am J Psychiatry.

